# Anion Exchange Membrane Based on Interpenetrating Polymer Network with Ultrahigh Ion Conductivity and Excellent Stability for Alkaline Fuel Cell

**DOI:** 10.34133/2020/4794706

**Published:** 2020-05-13

**Authors:** Lingping Zeng, Qian He, Yunchuan Liao, Shangyi Kuang, Jianchuan Wang, Wei Ding, Qiang Liao, Zidong Wei

**Affiliations:** ^1^School of Chemistry & Chemical Engineering, Chongqing University, 400044 Chongqing, China; ^2^School of Energy and Power Engineering, Chongqing University, 400044 Chongqing, China

## Abstract

A high-performance anion exchange membrane (AEM) is critical for the development of alkaline fuel cell. In this work, AEMs with an interpenetrating polymer network (IPN) are synthesized. An electron microscope clearly reveals a highly efficient “ion channel” network, which is constructed with a small amount of cation exchange groups. This specially designed ion channel leads to extraordinary hydroxide conductivity (e.g., 257.8 mS cm^−1^ at 80 °C) of IPN AEMs at moderate ion exchange capacity (IEC = 1.75 mmol g^−1^), as well as excellent long-term alkaline stability at harsh condition which showed that 81% of original conductivity can be retained after a long time for 1248 hours. Moreover, a remarkable peak power density of 1.20 W cm^−2^ (0.1 MPa backpressure) with nonprecious metal (FeNx-CNTs) as oxygen reduction reaction (ORR) catalyst in a fuel cell test was achieved. This work offers a general strategy to prepare high-performance AEMs based on IPN structure design.

## 1. Introduction

As one of the electrochemical energy storage and conversion technologies, fuel cells are expected to be one of the promising environmentally friendly power sources. In all kinds of fuel cells, alkaline anion exchange membrane fuel cells (AEMFCs) have attracted more and more attentions, since nonprecious catalyst can be adopted to reduce the cost, and the kinetic of oxygen reduction is faster, as well as less corrosion problems in alkaline condition. [1–6] As a crucial component in AEMFCs, anion exchange membranes (AEMs) segregate the fuel (anode) from oxidant (cathode) and provide an ion transport pathway simultaneously. [7] Therefore, high ion conductivity and excellent mechanical and chemical stability are essential for ideal AEMs. [8, 9] However, both of which have always been the two major challenges in AEMs up to date.

A facile way to improve the ion conductivity of AEMs is increasing the ion exchange capacity (IEC, i.e., the concentration of fixed ion groups in AEMs). However, high IEC value is usually accompanied by high dimensional swelling which leads to the deterioration of mechanical stability of AEMs. [10–12] In this case, crosslinking technology was used to suppress the undesirable excessive dimensional swelling. [13–17] Nonetheless, crosslinking of polymer chains could hinder the ion transport in reverse; thus, specific crosslinking method for each polyelectrolyte should be carefully designed to balance the dimensional swelling and ion conductivity. [18] Microphase separation is another way to prepare high-performance AEMs. [19–24] In this strategy, a small amount of cation exchange groups form an efficient continuous “ion channel” due to microphase separation, which facilities the anion conduction greatly. Therefore, with such a structural control method, high ion conductivity and mechanical stability can be obtained simultaneously. Similarly, an interpenetrating polymer network (IPN) has a “multiple continuous phase” structure, in which two or more phases are interlaced with each other. [25] For instance, Pan et al. prepared a semi-IPN AEM composed of a rigid polymer network and ion-conductive component, which exhibited outstanding mechanical strength (17.4 MPa) and flexibility (93.4% elongation), as well as good ion conductivity (67.7 mS cm^−1^ @ 80°C). [26] Other semi-IPN AEMs [14, 27–35] such as crosslinked quaternized poly(epichlorohydrin)/PTFE system were also reported to be helpful for the improvement of AEMs. Theoretically, with both the two polymer phases crosslinked, better mechanical strength of full IPN AEMs can be achieved. Such as an IPN AEM based on poly(vinyl alcohol) and polyethyleneimine was reported to possess much higher mechanical strength than semi-IPN AEMs and blended AEMs. [36] There have been several other literatures on IPN AEMs, yet the comprehensive properties of the reported membranes were ordinary which cannot meet the requirement of alkaline fuel cell. [37–39] Therefore, further efforts must be made to achieve excellent membrane properties with this strategy.

In this paper, crosslinked poly(vinyl alcohol)/crosslinked poly (vinyl benzyl-N-methyl piperidinium) IPN AEM was developed. The IPN structure of AEM was clearly demonstrated with an electron microscope, and extraordinary membrane conductivity and cell performance were achieved.

## 2. Results

### 2.1. Chemical Structure of the IPN AEMs

**Figure 1:**
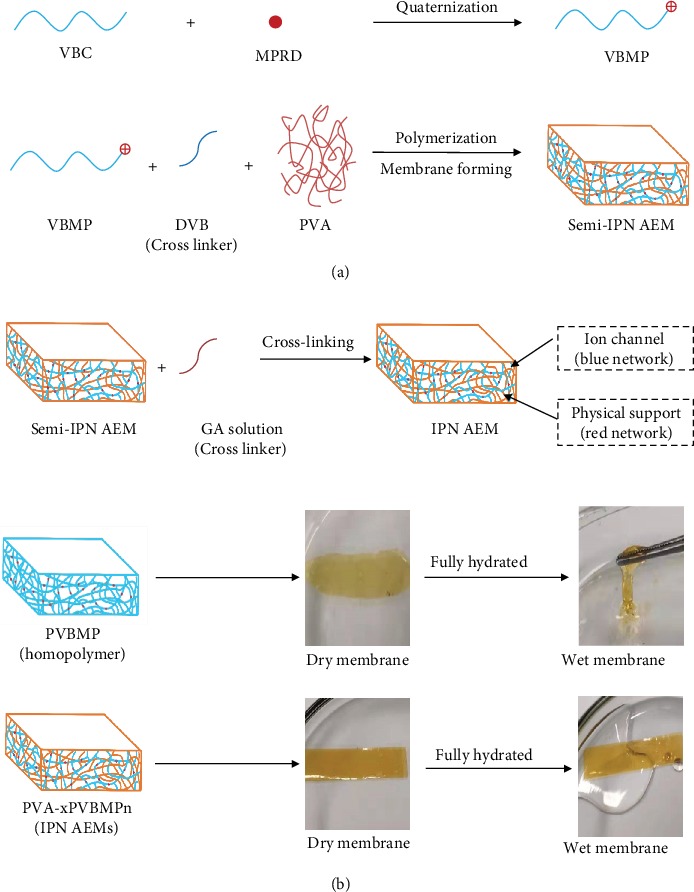
Fabrication process of IPN AEMs (a), digital photos of PVBMP and IPN AEMs in dry and fully hydrated state (b).

The fabrication procedure of IPN AEMs is shown in Figure 1. Quaternized vinylbenzyl-N-Methylpiperidine (QVBMP) monomer was synthesized by quaternization of VBC with N-Methylpiperidine first; then, the VBMP monomer and divinylbenzene (DVB, crosslinker) were in situ polymerized in poly(vinyl alcohol) (PVA) solution, forming a quaternized poly(vinylbenzyl-N-methyl piperidinium) (PVBMP) network which was interlaced with PVA molecules. As the polymerization and membrane forming were accomplished on a glass mold simultaneously, thus a semi-IPN membrane was prepared. At last, the semi-IPN membrane was converted to IPN membranes by crosslinking PVA molecules with glutaraldehyde (GA). There were two polymer networks in IPN AEM which were interlaced with each other. The first polymer network was a PVBMP network (blue one), functionalized with quaternized vinylbenzyl-N-methylpiperidine groups as ion transport paths. The second polymer network was a PVA network (red one), acting as physical support which endows the membrane good mechanical properties. As shown in the digital photos of Figure 1, the dry membrane of PVBMP homopolymer was quite brittle and totally lost its mechanical integration when fully hydrated. However, when interlaced with the PVA network, the as-prepared IPN AEMs showed excellent membrane forming ability and good mechanical properties.

**Figure 2:**
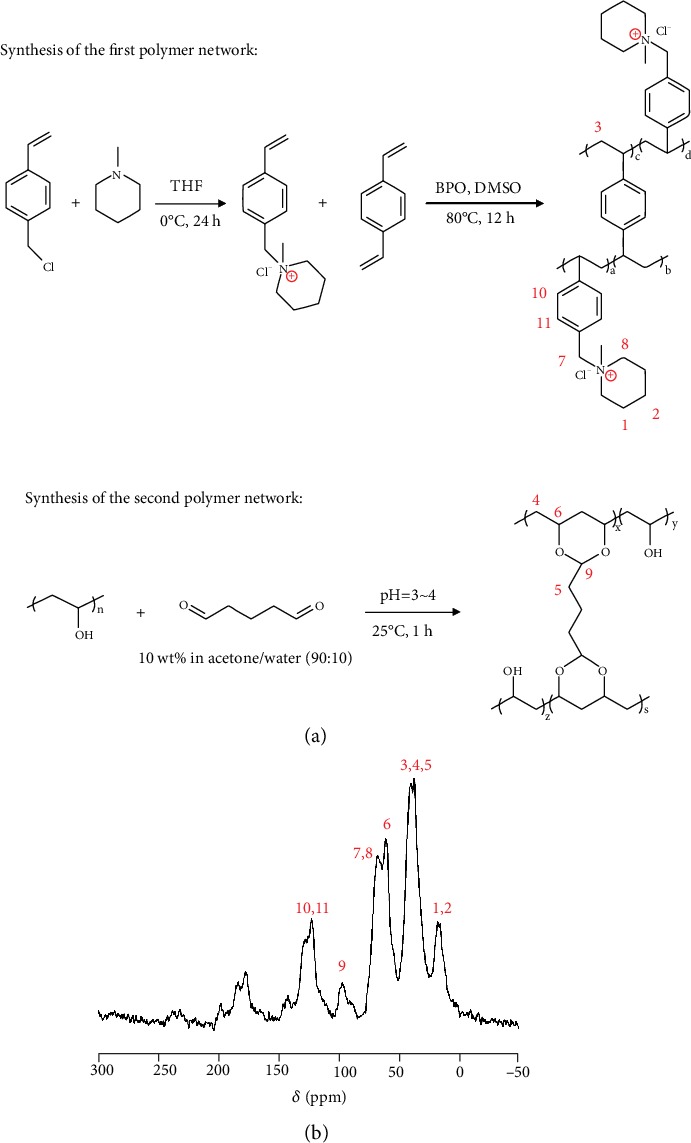
(a) Chemical synthesis of the first (crosslinked PVBMP) and second (crosslinked PVA) polymer networks. (b) Solid-state ^13^C NMR spectra of IPN AEM (PVA-1.8PVBMP, in Cl^−^ form).

The chemical synthesis of the two polymer networks is shown in Figure 2(a), the PVBMP network was synthesized by radical polymerization; its crosslinking degree was fixed to be 40% of DVB. The PVA network was synthesized by acetalation reaction; its crosslinking degree was kept with the same value for all membranes, by strictly fixing the acetalation reaction condition (GA concentration, pH value, temperature, time, etc.) the same. To study the chemical structure of IPN AEMs, the solid-state ^13^C NMR of PVA-1.8PVBMP was carried out, and the result is shown in Figure 2(b). The peaks at *δ* = 15.6 − 17.7 ppm were attributed to the CH_2_-CH_2_ (position 1,2) in cycloaliphatic of the piperidinium ring, the peaks at *δ* = 37.3 − 40.5 ppm were ascribed to the CH_2_-CH_2_ (positions 3, 4, and 5) of the PVA and PVBMP main chains as well as GA, and the peaks at *δ* = 60.5 ppm can be attributed to the C-O bond at position 6. [40] The benzyl carbon at position 7 overlapped with the cycloaliphatic carbon at position 8, which gave rise to signal at *δ* = 66.8 ppm. The signal at position 9 (*δ* = 96.5 ppm) indicated the acetalation structure induced by PVA crosslinking, and the peaks at *δ* = 122.8 − 128.1 ppm (positions 10 and 11) related to the carbons on the benzene ring. The characteristic peaks which arose from ^13^C NMR spectra verified the successful synthesis of IPN AEMs. Besides, FTIR spectra can also verify the chemical structure of IPN AEMs (Figure S1).

### 2.2. Physical Structure of the IPN AEMs

**Figure 3:**
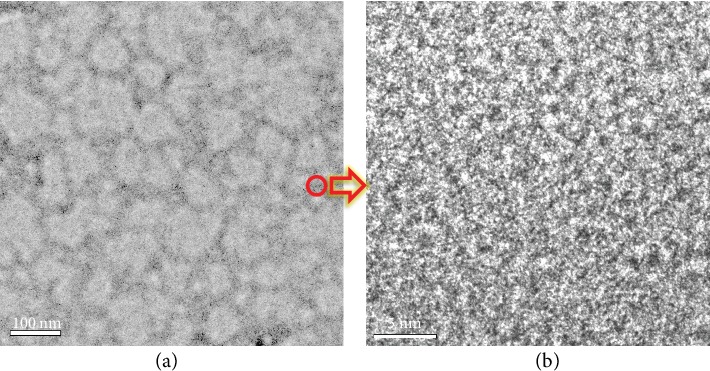
TEM images of IPN AEM (PVA-1.8PVBMP) at (a) low magnification and (b) the dark network area (red circle in (a)) at high magnification. The membrane was microtome sectioned to ultrathin slice and stained with phosphotungstic acid.

In order to verify the successful construction of the interpenetrating polymer network, the representative PVA-1.8PVBMP IPN AEM was directly embedded with epoxy resin, microtome sectioned to ultrathin slice and stained with phosphotungstic acid, thoroughly investigated by TEM. As shown in Figure 3(a), one might observe a recognizable dark network which was the PVBMP network, because the PVBMP phase was stained by phosphotungstic acid. While the rest of the picture (light area) belonged to the PVA phase, which was also a continuous phase (three-dimensional polymer network). Therefore, the PVBMP network was interpenetrated with the PVA network, confirming the successful construction of IPN AEM. The ion exchange group scattering was investigated further. The PVBMP network was magnified and is shown in Figure 3(b); the dark dots were ion exchange groups, which showed random distribution in the PVBMP phase. There was neither ion cluster nor microphase separation, which could be verified by SAXS results (Figure S2); no peak emerged in the range of *q* = 0.5 nm^−1^ to 7 nm^−1^ for all dry IPN AEMs, indicating there was no ordered structure in the range of *d*-spacing from 0.9 nm to 12.6 nm. Note that there seemed to be “nanophase separation” morphology in Figure 3(b); however, it might come from the sample preparation and observation in TEM. Compared with traditional AEM in which cationic groups are randomly distributed within the entire membrane, the cation groups of IPN AEMs in this work were confined in the PVBMP network, forming an efficient ion transport path; thus, excellent conductivity was expected.

### 2.3. Performance of the IPN AEMs

#### 2.3.1. Conductivity

**Figure 4:**
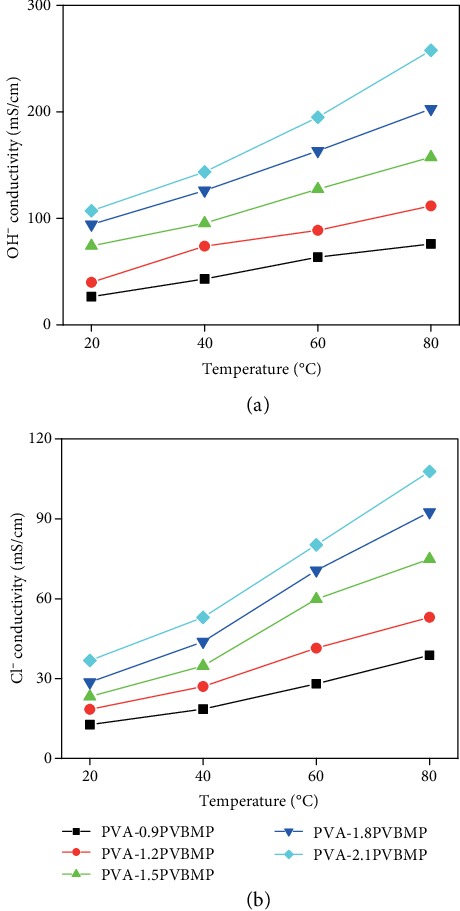
(a) Hydroxide and (b) chloride conductivities of PVA-*x*PVBMP at a fully hydrated state.

The in-plane hydroxide conductivities of IPN AEMs were measured with fully hydrated membranes, as a function of temperature. As shown in Figure 4(a), the OH^−^ conductivities of IPN AEMs were enhanced with the increasing PVBMP mass ratio in all range of temperature, which was attributed to the increase of the IEC value. Moreover, with temperature elevated, conductivities of all samples increased. The activation energies (*E*_a_) were calculated, by linear fitting of ln *σ* with 1/*T*, based on the Arrhenius equation (Figure S3). The *E*_a_ values for IPN AEMs were from 12.63 kJ mol^−1^ to 15.42 kJ mol^−1^, which was similar to the reported literature. [41] Overall, IPN AEMs exhibited excellent OH^−^ conductivities, at a moderate IEC value from 0.88-1.75 mmol g^−1^. For instance, PVA-2.1PVBMP (IEC = 1.75 mmol g^−1^) possessed extraordinarily high conductivity of 107.1 mS cm^−1^ at 20°C, which boosted to be an amazing value of 257.8 mS cm^−1^ at 80°C. Besides, to totally exclude the influence of carbon dioxide on testing, the Cl^−^ conductivity was also measured. As shown in Figure 4(b), although the Cl^−^ conductivity of all membranes were much lower than OH^−^ conductivity, which was because the mobility of Cl^−^ was only 39% of that of OH^−^, [42] Cl^−^ conductivity as high as 107.8 mS cm^−1^ at 80°C for PVA-2.1PVBMP was observed, verifying the excellent anion conductivity of the IPN AEMs. Above all, the conductivity achieved by the as-prepared IPN AEM was among the highest ones reported in recent years (see Table S1). The high conductivities of IPN AEMs were mainly attributed to the efficient ion conduction path (PVBMP network), which was constructed by IPN design. Besides, Dong et al. [43] reported a super high proton conductivity in Nafion nanofibers, attributing to the preferential alignment of interconnected ionic aggregates along the fiber axis direction. In this work, ion groups were confined in a quite small polymer network (only dozens of nanometers); there might similar promotion in ion conductivity.

#### 2.3.2. Mechanical and Thermal Properties

**Figure 5:**
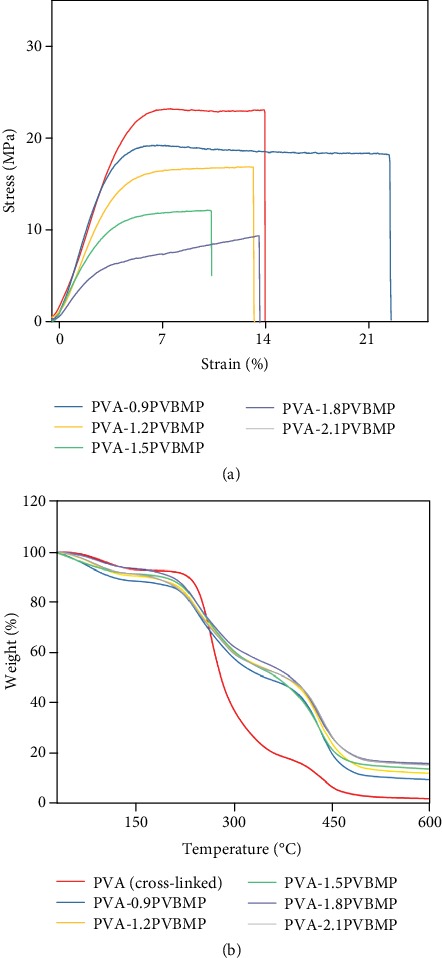
(a) Stress-strain curves of IPN AEMs. Membranes were fully hydrated. (b) TGA curves of IPN AEMs under N_2_ atmosphere.

In addition to high conductivity, good mechanical properties were also essential for AEM application. Therefore, fully hydrated IPN AEMs were tested, and the stress-strain curves of some IPN AEMs are shown in Figure 5(a). With PVBMP mass ratio increasing, the tensile strength of INPAEMs decreased. However, for PVA-2.1PVBMP, an acceptable tensile strength of 9.3 MPa could still be achieved. The highest tensile strength was up to 23.3 MPa, for PVA-0.9PVBMP (Table 1). Besides, the elongations at break of IPN AEMs were all beyond 10%, suggesting a good toughness which benefits for membrane operation. The good mechanical properties could be ascribed to the IPN structure in which two polymer networks were interlaced with each other, as well as physical crosslinking by PVA crystals (Figure S4). The tensile strength for all IPN AEMs are listed in Table 1, as well as IEC, water uptake, swelling ratio, conductivity, and activation energy.

The thermal stability of IPN AEMs was excellent. As shown in Figure 5(b), for crosslinked PVA (control sample, fabrication process was in the SI materials) and IPN AEMs, there were three weight loss stages at around 100°C, 250°C, and 450°C. The first loss can be ascribed to the water evaporation, the second one was related to the cationic or hydroxyl group decomposition, and the third one was the degradation of polymer backbone. Since fuel cell usually operated at 60-80°C, thus, the IPN AEMs can work stably under working condition.

#### 2.3.3. Chemical Stability

**Figure 6:**
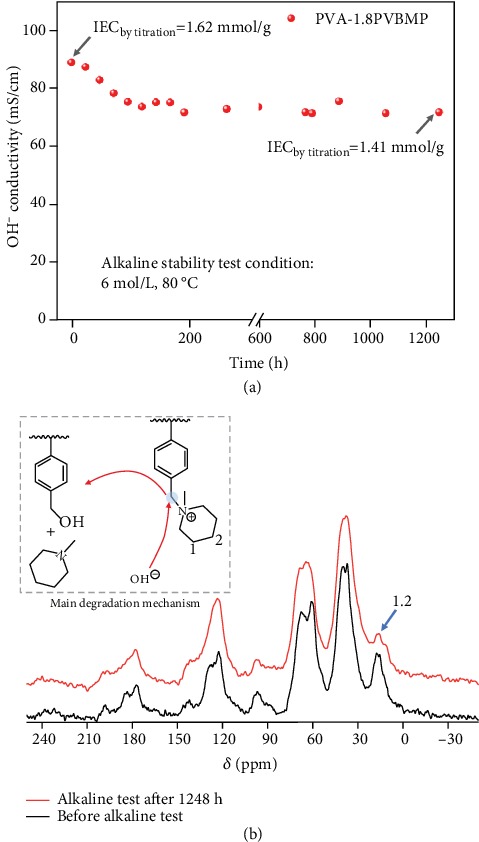
(a) Alkaline stability tests of IPN AEM. The sample was immersed in 6 mol L^−1^ NaOH at 80°C, and the hydroxide conductivities were measured at 20°C. (b) Solid-state ^13^C NMR spectra of PVA-1.8PVBMP before the alkali resistance test (black) and after the alkali resistance test (red).

The alkaline stability of IPN AEM was carried out with a harsh condition, that was soaking in 6 mol L^−1^ NaOH at 80°C; then, the hydroxide conductivity was recorded at intervals (at 20°C). As shown in Figure 6(a), the OH^−^ conductivity of fresh PVA-1.8PVBMP was 89.3 mS cm^−1^ @20°C, which decreased to 75 mS cm^−1^ @20°C and almost kept constant from then on. After a long time for 1248 h, the membrane still possessed a very high conductivity of 71.9 mS cm^−1^ @20°C, showing only a 19% decrease in OH^−^ conductivity. Accordingly, the IEC of the membrane decreased from 1.62 mmol g^−1^ to 1.41 mmol g^−1^, with a small loss of 13%. One may have concern of NaOH doping into the PVA phase which resulted in the residual OH^−^ conductivity; thus, the Na^+^ content in the sample (after alkaline stability) was determined by an ICP test. The result demonstrated that the Na^+^ content was as extremely low as 2.67 × 10^−4^ g g^−1^, which contributed only 0.012 mmol g^−1^ to the IEC of the sample after the alkaline stability test. This result clearly proved that there was hardly NaOH doped in the PVA phase; thus, the concern of NaOH doping can be eliminated. On the other hand, the solid-state ^13^C NMR spectra of PVA-1.8PVBMP before and after the alkali resistance test were investigated. As shown in Figure 6(b), the ^13^C NMR spectrum of the sample after the alkaline test was similar to that of the sample before the alkaline test, implying the chemical structure of the IPN AEM was retained well. Therefore, it was the highly alkaline resistance essence of IPN AEM rather than NaOH doping which led to the high residual OH^−^ conductivity after a long-time alkaline stability test. Technically, there was indeed a small quantity of cation exchange groups decomposed judging from the ^13^C NMR spectra, which resulted in the minor decrease in conductivity. As shown in Figure 6(b), compared with PVA-1.8PVBMP before the alkali stability test, the peak intensity at positions 1 and 2 (ascribed to CH_2_-CH_2_ in cycloaliphatic of piperidinium ring) of PVA-1.8PVBMP after the alkali stability test slightly decreased, implying the piperidinium ring came off the polymer backbone. Therefore, the main decomposition mechanism for IPN AEMs is the nucleophilic substitution at *α* carbon (benzyl methylene, see insert of Figure 6(b)).

To explain the high alkaline resistance of IPN AEMs, the first reason should be the good alkaline stability of the piperidinium ring, [44] and the second one might be the high water uptake resulting in high solvation of cationic groups which reduced the attack ability of OH^−^. [45] For the third reason, we speculated that there was a “passivation layer” formed (see Figure S5), and the OH^−^ was confined in the interphase of PVA and PVBMP phases; thus, it was hard to go through the “passivation layer” and further attacked the cationic groups inside the PVBMP phase, which might be able to explain the phenomenon of the conductivity which decreased no more after 200 h.

#### 2.3.4. Single Cell Performance

**Figure 7:**
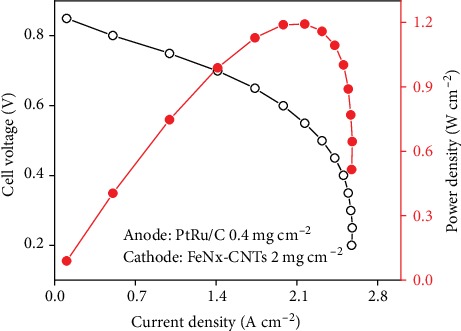
H_2_/O_2_ fuel cell performance of PVA-1.8PVBMP at 60°C; pure hydrogen and oxygen were supplied to the anode and cathode at a flow rate of 400 mL min^−1^. Solid circle and hollow circle were power density and cell voltage, respectively, with 0.1 MPa backpressure.

As one of the biggest advantages of AEMFCs, nonprecious metal catalyst can be used. Thus, FeNx-CNTs catalyst (see the synthesis and properties as Figure S6) was adopted as ORR catalyst for H_2_/O_2_ fuel cell performance, with an ~40 *μ*m PVA-1.8PVBMP membrane, and the data are demonstrated in Figure 7. The water uptake of the as-made IPN AEMs was relatively high; in order to improve the oxygen transfer efficiency, gas diffusion electrode (GDE) with a better gas diffusion channel was adopted to make membrane electrode assembly (MEA); PtRu/C and FeNx-CNTs were sprayed onto anode (0.4 mg cm^−2^) and cathode (2 mg cm^−2^) carbon paper, respectively, both with 20wt% quaternized poly(2,6-dimethyl-1,4-phenylene oxide (QPPO) [46] (see the detail preparation and characterization as Figure S7 and Table S2) as ionomer. The open-circuit voltage of fuel cell was 0.87 V, indicating excellent membrane integrity with quite low gas permeability. After a backpressure of 0.1 MPa was applied, the peak power density reached as high as 1.20 W cm^−2^. As listed in Table S1, this result even surpassed the highest peak power density (1.1 W cm^−2^) reported with precious metal-free cathode reported recently. [47] The dramatic drop in power density after a high current density of 2.2 A cm^−2^ was ascribed to the catalyst layer flooding. [48] With the current density large increased, the electroreaction speed increased, thus much more water generated; if the water cannot be removed from the catalyst layer, the water molecules will block the way of oxygen to the surface of catalyst. As a result, the electroreaction speed slowed down, which resulted in power density drop. Thus, IPN AEMs with less water uptake (more hydrophobic) should be developed in the future to further improve the cell performance.

## 3. Conclusions

In conclusion, crosslinked poly (vinyl alcohol)/crosslinked poly (vinyl benzyl-N-methyl piperidinium) IPN AEM was successfully synthesized. The electron microscope clearly revealed an IPN structure, in which one ion conductive polymer network was interlaced with the other nonconductive polymer network, which resulted in a highly efficient “ion channel” constructed. With this structure, outstanding hydroxide conductivity of IPN AEMs with a moderate IEC value was achieved. For instance, PVA-2.1PVBMP exhibited an OH^−^ conductivity of 257.8 mS cm^−1^ (@80°C) with an IEC value of 1.75  mmol g^−1^, while possessed a good tensile strength of 9.3 MPa (wet membrane). The alkaline stability of the IPN AEMs was excellent as well; 81% of original conductivity can be retained after a long time for 1248 h. With nonprecious FeNx-CNTs as ORR catalyst, the cell performance based on the as-prepared IPN AEM demonstrated a peak power density up to 1.20 W cm^−2^ at 60°C (0.1 MPa backpressure). Future work should be the reducing of the hydrophilicity of IPN AEMs to avoid flooding. This work offered a general strategy to prepare high-performance AEMs base on the IPN structure.

## 4. Materials and Methods

### 4.1. Materials

4-vinylbenzyl chloride (VBC), polyvinyl alcohol (PVA) (Mw = 80000 g mol^−1^, 99%^+^ hydrolyzed) and divinylbenzene (DVB) were purchased from Sigma-Aldrich, N-Methylpiperidine (MPRD) and benzoyl peroxide (BPO) were supplied by Adamas Reagent Co., Ltd., and glutaraldehyde (GA, 50%) was obtained from Chengdu Kelong Reagent Co., Ltd. VBC and DVB have their inhibitors removed before used; BPO was recrystallized with methanol before used. Other chemicals were used as received.

### 4.2. Methods

First, vinylbenzyl-N-methylpiperidinium (VBMP) was synthesized by stirring the mixture of VBC with MPRD (1 : 1.5 molar ratio) for 24 h at 0°C. The resultant product was washed with ether for several times, dried at room temperature. After that, 0.5 g VBMP, 0.2 g DVB, and 0.0035 g BPO were added to PVA solution (2% *w*/*v* in DMSO), stirred for 0.5 h to obtain homogenous solution. Next, the solution was poured onto a clean and flat glass plate, heated for 12 h (at 80°C, ambient environment) followed by vacuum drying at 60°C for 24 h. As a result, semi-IPN AEMs were prepared. The semi-IPN AEMs were further converted to IPN AEMs by crosslinking of PVA molecules in a typical way: dried semi-IPN AEMs were soaked in glutaraldehyde (GA) solution (20 g acetone, 2 g GA solution, and 0.04 g hydrochloric acid, pH < 7) at 25°C for 1 h. Finally, crosslinked poly(vinyl alcohol)/crosslinked poly(vinylbenzyl-N-methylpiperidinium) IPN AEMs were fabricated. Samples were designated as PVA-*x*PVBMP: *x* represented the mass ratio of VBMP with PVA; for example, in PVA-0.9PVBMP membrane, PVBMP/PVA = 0.9 *w*/*w*. The fabrication of the PVA comparison sample for FTIR and TGA characterization was summarized in the SI materials.

### 4.3. Characterization

The chemical structures of IPN AEMs were characterized by Fourier Transform Infrared spectroscopy (FTIR) spectra using a Nicolet iS50 system with the resolution of 4 cm^−1^; samples were ground and measured with transmission mode. The IPN structure and cationic group scattering were investigated with a transmission electron microscope (TEM, FEI Talos F200S G2), using an acceleration voltage of 200 kV; samples were microtome sectioned to ultrathin slice and stained with phosphotungstic acid. Tensile measurements were performed using a MTS Tensile Tester (E44.104), at a rate of 5 mm min^−1^ and room temperature; all samples were fully hydrated. Solid-state ^13^C NMR spectra were obtained by an Agilent 600 M spectrometer at 600 MHz. Wide-angle X-ray diffraction data were collected with an XRD-6000 using Cu Kr radiation at a scanning rate of 2 min^−1^. Small-angle X-ray scattering (SAXS) measurements were conducted on an Xeuss 2.0 SAXS/WAXS system, with the wavelength of 0.154 nm, operating at 50 kV and 0.6 mA; dry membranes were used. The inductively coupled plasma (ICP) measurements were conducted on an iCAP 6000 Series spectrometer to determine the Na^+^ content in IPN AEMs after the alkaline stability test. The thermal stability was characterized with a thermogravimetric analysis (TGA-Q500, TA Instruments, New Castle, DE, USA), heated from room temperature to 600°C under N_2_ atmosphere at a rate of 10°C min^−1^. In-plane hydroxide and chloride conductivities of AEMs were measured by two-point probe AC impedance spectroscopy, using an electrochemical interface (Solartron AAnalytical 1287) in combination with an impedance/gain-phase analyzer (Solatron 1260), at a frequency range from 1 to 10^6^ Hz. A picture of the testing fixture was showed in Figure S8 in the SI. The membranes were measured at fully hydrated condition in the longitudinal direction with the whole testing fixture immersed in ultrapure water. The Cl^−^-form membranes were converted to OH^−^ form by being immersed in 1 mol L^−1^ NaOH for 48 h, followed by washing thoroughly with deionized water to remove any residual NaOH. The Cl^−^-form membranes were measured with the as-prepared membranes, without any treatment in NaCl solution. In order to reduce the impact of carbon dioxide on hydroxide conductivity testing, the samples were washed and mounted quickly as much as possible, to avert exposing in the air for a long time. Finally, *σ* can be calculated from the following equation:

(1) $$\begin{array}{c} \sigma =\frac{L}{R\times A}, \end{array}$$

where *R* is the resistance value (*Ω*) of the membrane, *L* is the distance between the two electrodes, and *A* is crosssectional area of the membrane.

Activation energy (*E*_a_) of ionic conductivity can be determined with Arrhenius relationship between the conductivity and temperature, which can be expressed as follows:

(2) σT=σ0e,−Ea/RT

where *σ*_*T*_ is the ionic conductivity at temperature *T*, *σ*_0_ is the Arrhenius constant, and *R* is gas constant.

The ion exchange capacity (IEC) was determined by the titration method. The as-prepared original membranes (about 0.2 g) in Cl^−^ form were immersed in 40 mL NaNO_3_ (0.1 mol L^−1^) for 48 h at 40°C and titrated by AgNO_3_ (0.01 mol L^−1^, the concentration was calibrated before use), with potassium chromate as indicator. For the alkaline stability test sample, the sample was pretreated with 1 mol L^−1^ NaCl solution to converted the OH^−^ to Cl^−^. The IEC values were calculated from the equation:

(3) $$\begin{array}{c} \text{IEC}=\frac{0.01\times v_{{\text{AgNO}}_{3}}}{w_{\text{dry}}\left({\text{Cl}}^{‐}\right)}, \end{array}$$

where *v*_AgNO3_ is the volume of AgNO_3_ consumed in titration and *w*_dry_(Cl^−^) is the weight of the Cl^−^-form membrane after vacuumed-dried.

For water uptake (WU) and swelling ratio (SR), membranes were dried under vacuum at 60°C for 24 h; then, the mass and length were recorded. After that, the dried membrane was immersed in DI water for 24 h to achieve full hydration and wiped water out with tissue paper; the mass and length were quickly measured. The WU can be calculated from the equation:

(4) $$\begin{array}{c} \text{WU}\left(\%\right)=\frac{W_{\text{wet}}-W_{\text{dry}}}{W_{\text{dry}}}\times 100\%, \end{array}$$

where *W*_wet_ and *W*_dry_ are the mass of the hydrated sample and dried sample, respectively.

The SR can be determined from the following:

(5) $$\begin{array}{c} \text{SR}\left(\%\right)=\frac{l_{\text{wet}}-W_{\text{dry}}}{W_{\text{dry}}}=100\%, \end{array}$$

where *l*_wet_ and *l*_dry_ are the length of hydrated sample and dried sample, respectively.

To evaluate the cell performance with our IPN AEMs, membrane electrode assembly (MEA) was made by a gas diffusion electrode (GDE) method. FeNx-CNTs or PtRu/C catalysts (60 wt% in metal content) were mixed with QPPO ionomer (IEC = 1.64 mmol g^−1^, hydroxide conductivity = 33.0 mS cm^−1^@20°C, and water uptake = 22.3%), ultrasonicated to yield inks consisting of 20 wt% ionomer and 80 wt% catalyst. Then, FeNx-CNTs ink and PtRu/C ink were sprayed onto the cathode and anode carbon papers (the electrode area was 1 cm^−2^). The metal loading in anode and cathode were 0.4 mg cm^−2^ and 2 mg cm^−2^, respectively. The prepared anode carbon paper, cathode carbon paper, and a PVA-1.8PVBMP membrane (~40 *μ*m) were immersed in 1 mol L^−1^ NaOH for 24 h to convert the halogen anion to hydroxide, followed by washing with DI water. Then, the anode and cathode carbon papers were positioned on the two sides of the membrane to make MEA. Single-cell fuel tests were carried out with a fuel cell test station (850e Multi Range, Scribner Associates Co.); pure humidified hydrogen and oxygen were supplied to the anode and cathode at a flow rate of 400 mL min^−1^, with 0.1 MPa backpressure.

## Figures and Tables

**Table 1:** IEC, water uptake, in-plane swelling ratio, through-plane swelling ratio, strength, conductivity, and activation energy of IPN AEMs.

Sample	IEC^a^ (mmol g^−1^)	WU^b^ (%)	In-plane SR^b^ (%)	Through-plane SR^b^ (%)	Strength^c^ (MPa)	*σ*_Cl_^−^ @80°C (mS cm^−1^)	*σ*_OH_^−^ @80°C (mS cm^−1^)	*E*_a_ (kJ Mol^−1^)
PVA-0.9PVBMP	0.88	114.2	22.5	33.3	23.3	38.8	76.0	15.42
PVA-1.2PVBMP	1.01	102.3	20.3	25.5	19.5	53.0	111.7	14.62
PVA-1.5PVBMP	1.50	89.3	17.6	23.6	17.5	74.9	157.5	13.72
PVA-1.8PVBMP	1.62	78.5	15.4	19.2	12.4	92.5	202.8	12.97
PVA-2.1PVBMP	1.75	67.5	11.0	15.5	9.3	107.8	257.8	12.63

^a^Measured by silver nitrate titration, ^b^measured at 20°C, ^c^measured with fully hydrated membrane.

## Data Availability

All data needed to evaluate the conclusions in the paper are present in the paper and the Supplementary Materials. Additional data related to this paper may be requested from the authors.

## Supplementary Materials

Supplementary Materials Figure S1: FTIR spectra of IPN AEMs and crosslinked PVA sample. Figure S2: SAXS data of crosslinked PVA and IPN AEMs samples; dry membranes were measured. Figure S3: the Arrhenius plots for calculation of apparent activation energy. Table S1: comparison of reported high-performance AEMs and this work. Figure S4: XRD patterns of crosslinked PVA and IPN AEMs. Figure S5: Proposed reason for the high alkaline resistance of IPN AEMs. Figure S6: SEM images of nanotubes-like FeNx-CNTs at (a) low magnification and (b) high magnification (c) and (d) TEM images of the FeNx-CNTs. (e) Polarization curves of FeNx-CNTs and commercial Pt/C in oxygen-saturated 0.1 M KOH at a rotating speed of 1600 rpm with a sweep rate of 10 mV s^−1^. Figure S7: the chemical structure of QPPO ionomer used and its 1H NMR spectrum. Table S2: properties of QPPO ionomer. Figure S8: the conductivity testing fixture.
