# Supplementary material for: Anion Exchange Membrane Based on Interpenetrating Polymer Network with Ultrahigh Ion Conductivity and Excellent Stability for Alkaline Fuel Cell
Source: Research (Wash D C). 2020 May 13;2020:4794706. doi: 10.34133/2020/4794706 (PMC7243038; doi:10.34133/2020/4794706)
Supplement: Supplementary Materials — Figure S1: FTIR spectra of IPN AEMs and crosslinked PVA sample. Figure S2: SAXS data of crosslinked PVA and IPN AEMs samples; dry membranes were measured. Figure S3: the Arrhenius plots for calculation of apparent activation energy. Table S1: comparison of reported high-performance AEMs and this work. Figure S4: XRD patterns of crosslinked PVA and IPN AEMs. Figure S5: Proposed reason for the high alkaline resistance of IPN AEMs. Figure S6: SEM images of nanotubes-like FeNx-CNTs at (a) low magnification and (b) high magnification (c) and (d) TEM images of the FeNx-CNTs. (e) Polarization curves of FeNx-CNTs and commercial Pt/C in oxygen-saturated 0.1 M KOH at a rotating speed of 1600 rpm with a sweep rate of 10 mV s−1. Figure S7: the chemical structure of QPPO ionomer used and its 1H NMR spectrum. Table S2: properties of QPPO ionomer. Figure S8: the conductivity testing fixture. [file 4794706.f1.zip › 4794706.f1/Supplementary Materials.docx]

Supplementary Materials

**
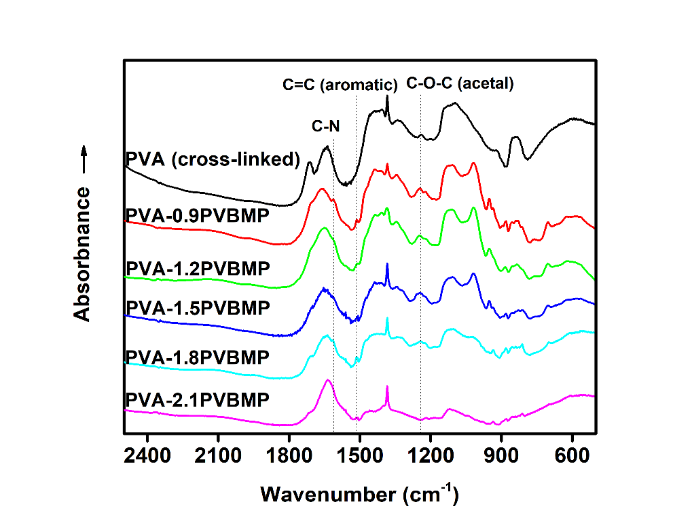
**

**Figure S1.** FTIR spectra of IPN AEMs and cross-linked PVA sample.

FTIR spectra with transmission mode was demonstrated in Figure S1 as supplementary evidence of the successful synthesis of IPN AEMs. Compared with cross-linked PVA sample, IPN AEMs including PVA-0.9PVBMP, PVA-1.2PVBMP, PVA-1.5PVBMP, PVA-1.8PVBMP and PVA-2.1PVBMP demonstrated characteristic signal at 1516 cm^-1^, which were ascribed to C=C bond from aromatic ring.[1] The weak signal at 1608 cm^-1^ for IPN AEMs were attributed to C-N bond from piperidinium. [2] The existence of C=C and C-N bonds suggesting the successful synthesis of PVBMP network. For IPN AEMs and cross-linked PVA, there was a weak signal around 1245 cm^-1^ which could be assigned to the C-O-C bond from acetal structure, [3] implying the PVA network was successfully fabricated.


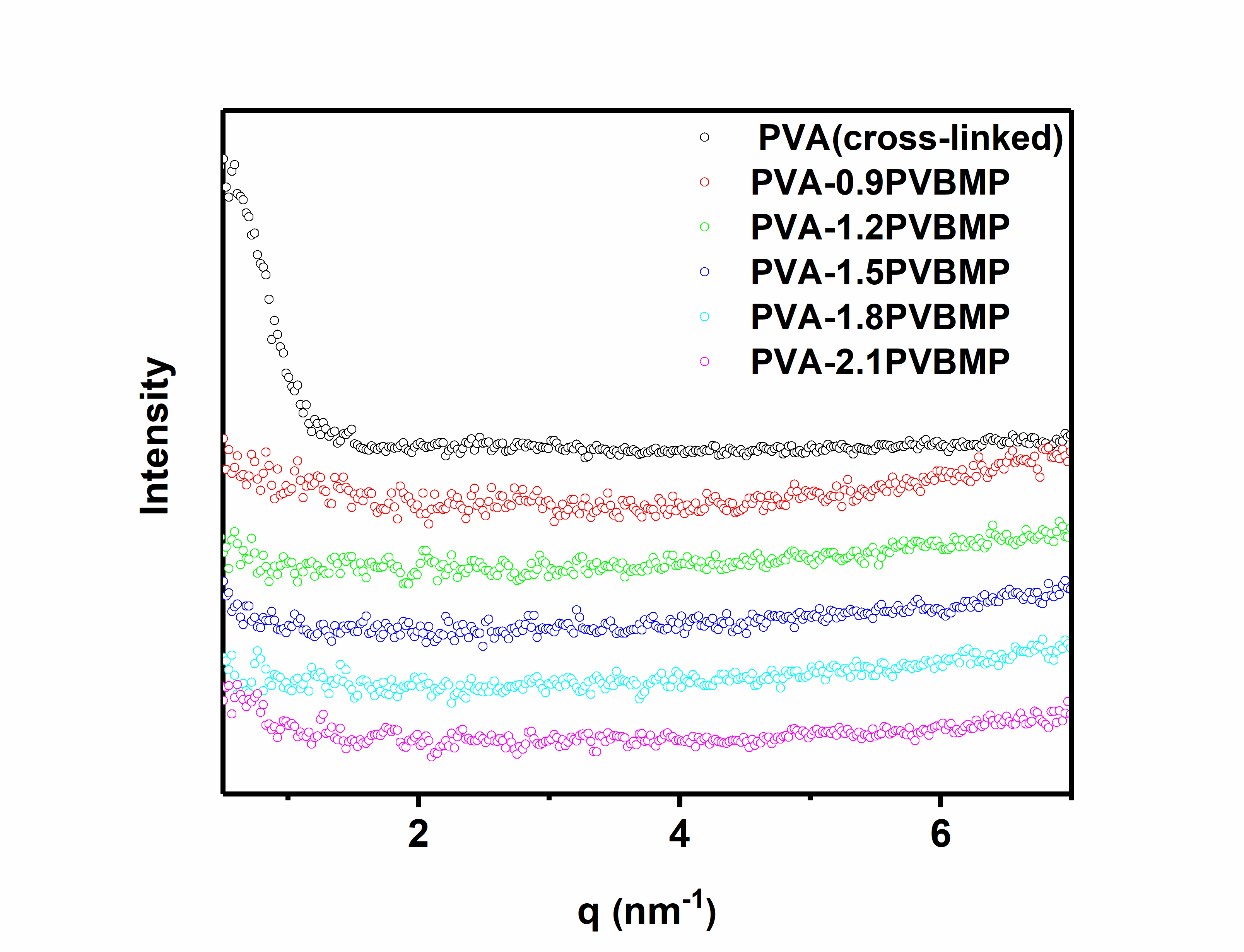


**Figure S2.** SAXS data of cross-linked PVA and IPN AEMs samples, dry membranes were measured.

**
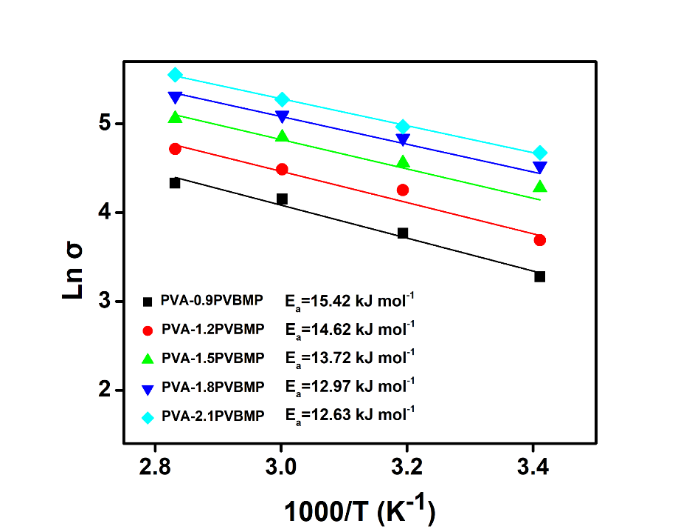
**

**Figure S3.** The Arrhenius plots for calculation of apparent activation energy.

**Table S1.** Comparison of reported high performance AEMs and this work.

| **Sample** | **Type** | **IEC**  **(mmol g^-1^)** | **Tensile strength (MPa)** | **σ_OH_^-^**  **(mS cm^-1^)** | **σ_Cl_^-^**  **(mS cm^-1^)** | **H_2_/O_2_ fuel cell peak power density**  **（W cm^-2^)** | **Ref.** |
| --- | --- | --- | --- | --- | --- | --- | --- |
| IPN AEMs | IPN | 1.75 | 9.3 | 257.8 (@80 ℃) | 107.8 (@80 ℃) | 1.2  (Anode: PtRu/C  Cathode: FeNx-CNTs Backpressure: 0.1 MPa) | This work |
| Tokuyama A201 | —— | 1.8 |  | 42  (@23 ℃) | —— | 0.12  (Anode: Pt/C  Cathode: Pt/C) | 4 |
| AAEM_3_**_d-e.2OH-_** | Comb-shaped polymer | 0.68 | —— | 189 (@90 ℃) | 122 (@90 ℃) | —— | 5 |
| PTPipQ1 | Poly(arylene piperidinium) | 2.42 | —— | 89  (@80 ℃) | —— | —— | 6 |
| S70P30 | N-Spirocyclic quaternary ammonium ionenes polymer | —— | —— | 120 (@90 ℃) | —— | —— | 7 |
| QPE-X16Y11 | Block copolymer | 1.93 | 13.0 | 144 (@80 ℃) | —— | 0.3  (Anode: Ni-Co  Cathode: Ni-Co  Backpressure: 0.02 MPa) | 8 |
| sp-PBI | Sandwiched-porous membrane | —— | 66.6 | 105 (@80 ℃) | —— | 0.54  (Anode:PdNWs/PBI/rGO  Cathode:PdWs/PBI/rGO  Backpressure: 0.2 MPa) | 9 |
| LDPE-BTMA AEM | Radiation-grafted polymer | 2.87 | 30 | 145 (@80 ℃) | 76 (@80 ℃) | 1.05  (Anode: PtRu/C  Cathode: N-C-CoO_x_  Backpressure: ~ 0.1 MPa) | 10 |
| OBImPPO-2.1 | Rotatable-spacer side-chains polymer | 2.1 | 5.8 | 65  (@60 ℃) | —— | 0.44  (Anode: PtRu/C  Cathode: Pt/C  Backpressure: 0 MPa) | 11 |
| aQAPS-S8 | Micro-phase separation polymer | 1.01 | 22.5 | 110 (@80 ℃) | —— | 1.0  (Anode: PtRu/C  Cathode: Pt/C  Backpressure: 0.1 MPa) | 12 |
| X80Y40C6 | Comb-shaped polymer | 2.35 | —— | 200 (@80 ℃) | 10.9 (@RT) | —— | 13 |
| E-5 | Radiation-grafted polymer | 2.13 | 27 | —— | 68 (@80 ℃) | 1.16  (Anode: PtRu/C  Cathode: Pt/C  Backpressure: 0.1 MPa) | 14 |
| T25NC6NC5N | multi-cation side chain polymer | 2.87 | —— | 99(@RT) | 34(@RT) | —— | 15 |
| RC-QPPO-2.13 | Cross-linked polymer | 2.13 | 23.14 | 69.6  (@80 ℃) | 30.3  (@80 ℃) | 0.15  (Anode: PtRu/C  Cathode: Pt/C  Backpressure: 0 MPa) | 16 |
| aQAPS-S8 | Micro-phase separation polymer | 1.01 | 22.5 | 110 (@80 ℃) | —— | 1.1  (Anode: PtRu/C  Cathode: Mn-Co  Backpressure: 0.1 MPa) | 17 |
| M20C9N6NC5N | Micro-phase separation polymer | 2.41 | —— | 201 (@80 ℃) | 21(@RT) | 0.94  (Anode: PtRu/C  Cathode: Pt/C  Backpressure: 0.05 MPa) | 18 |
| F20C9N | Micro-phase separation polymer | 1.12 | —— | 91  (@80 ℃) | 9(@RT) | 1.01  (Anode: PtRu/C  Cathode: Pt/C  Backpressure: 0.05 MPa) | 19 |
| HDPE-AEM | Radiation-grafted polymer | 2.44 | 35 | 214 (@80 ℃) | —— | 2.55  (Anode: PtRu/C  Cathode:Pt/C  Backpressure: 0.1 MPa) | 20 |


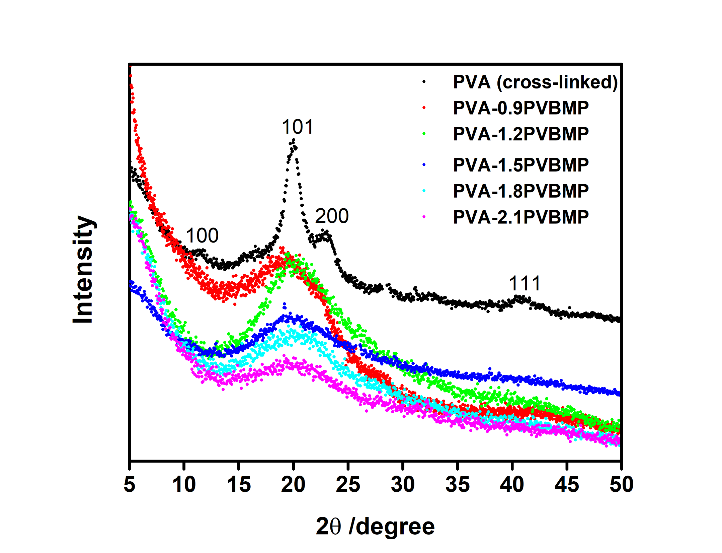


**Figure S4.** XRD patterns of cross-linked PVA and IPN AEMs.

In XRD patters, cross-linked PVA sample exbihited peaks at 2θ=11.5°, 19.9°, 22.8° and 40.7°, which were ascribed to 100, 101, 200 and 111 face of PVA crystals respectively. [21] For IPN AEMs, the signal at 19.9° were still observed, indicating that there were crystal structure induced by PVA in IPN AEMs, which may lead to enhanced mechanical properties by physical cross-linking with PVA crystals. Compared with cross-linked PVA, the peaks of IPN AEMs were much broader, because the PVA crystalization were disturbed by PVBMP molecules, resulted in more inperfect crystals.

**Preparation of cross-linked PVA sample (for TGA and FTIR control samples)**

0.5 g PVA was dissolved in 40 mL DMSO, stirred for 0.5 h to obtain PVA solution. Next, the solution was poured onto a clean and flat glass plate, heated at 80 ^o^C for 12 h, followed by vacuum drying at 60 ^o^C for 24 h. Then, the as-prepared PVA membrane was soaked in glutaraldehyde (GA) solution (20 g acetone, 2 g GA solution and 0.04 g hydrochloric acid, pH<7) for 1 h. Finally, PVA (cross-linked) sample were fabricated.


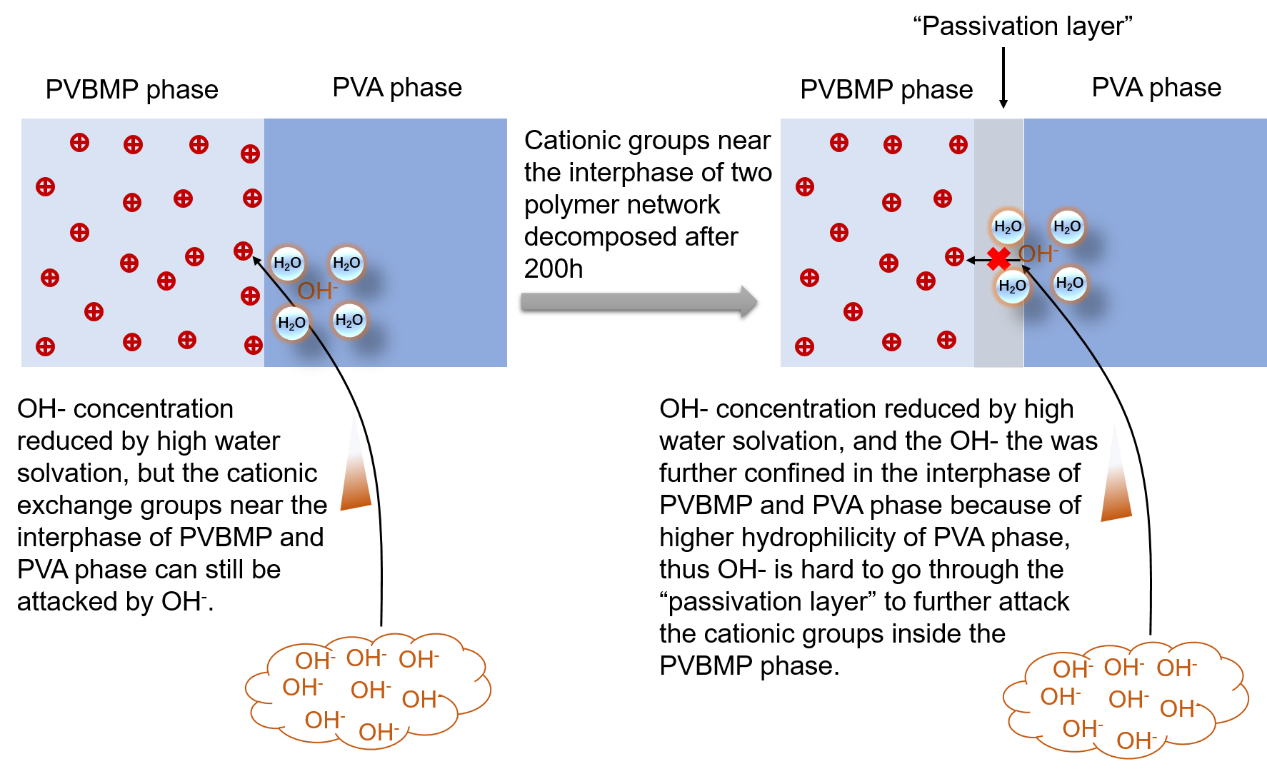


**Figure S5.** Proposed reason for the high alkaline resistance of IPN AEMs.

**Preparation of non-precious FeNx-CNTs catalyst**

2.974 g Zn(NO_3_)_2_·6H_2_O and 1.2247 g FeC_6_H_5_O_7_ were dissolved in 100 mL deionized water under stirring for 4 h at 60 ℃. Then 6.568 g 2-methylimidazole was mixed with 100 mL deionized water and subsequently added to the above mixture and stirring for another 24 h. After transferred to ice bath and kept for 30 min, a certain amount of pyrrole (Py) monomer was added and kept stirring for 12 h. Then aqueous solution of ammonium peroxydisulfate (initiator) was slowly added and kept stirring for 24 h. The product was then filtered and washed with deionized water and ethanol, followed by drying under 60 ℃ for 24 h to obtain a light grey Fe-ZIF@PPy powder. At last, Fe-ZIF@PPy was carbonized at 950 ℃ for 2 h under N_2_ flow with a ramping rate of 5 °C min^-1^. The resulting catalyst was labelled as FeNx-CNTs.


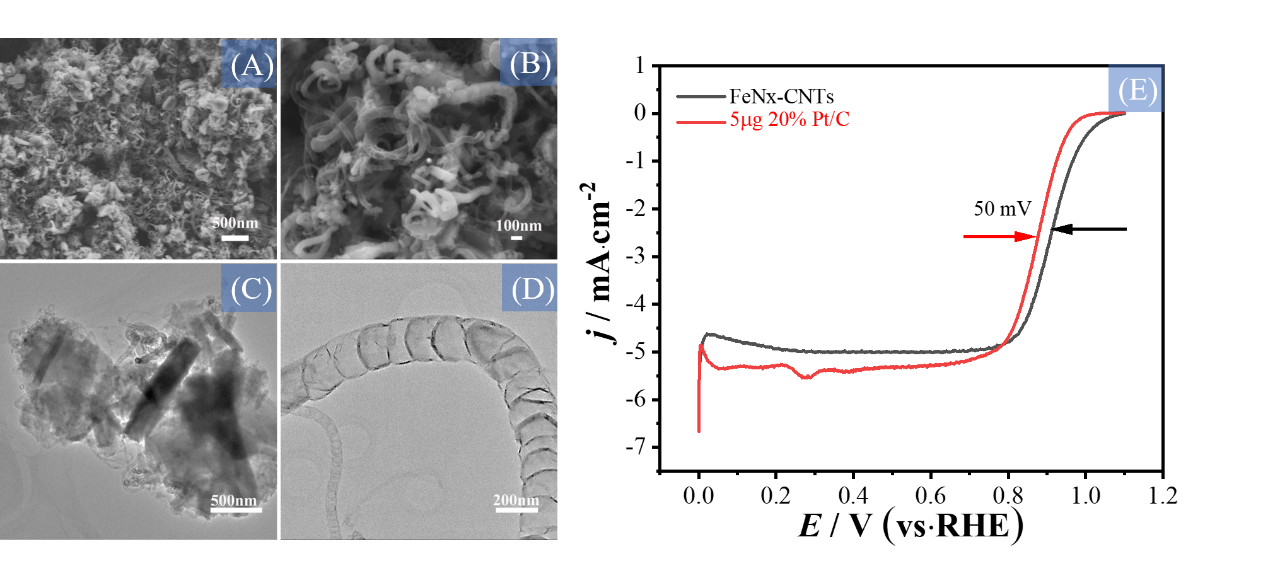


**Figure S6.** SEM images of nanotubes-like FeNx-CNTs at (a) low magnification and (b) high magnification; (c) and (d) TEM images of the FeNx-CNTs; (e) Polarization curves of FeNx-CNTs and commercial Pt/C in O_2_-saturated 0.1M KOH at rotating speed of 1600 rpm with a sweep rate of 10 mV/s.

The morphological and microstructure of electro-catalysts were investigated from the field emission scanning electron microscopy (FESEM) and transmission electron microscopy (TEM). As shown in Figure S6 (A,B,C,D), the FeNx-CNTs catalyst exhibited a carbon nanotubes (CNTs) predominant morphology with a diameter from 50-200 nm, which might arise from the high temperature pyrolysis turning Fe ions/cluster into Fe metal nano-catalysts, these Fe nano-catalysts then further catalyzed most of the organic units to form CNTs. The CNTs structure would increase the conductivity and specific surface area of the catalyst, thus enhancing the utilization of active sites. Such a catalyst with enriched active sites, efficient mass transportation pathway and optimal electrical conductivity, was expected to boost the electrocatalytic performance in anion exchange membrane fuel cells.

To investigate the electrocatalytic activities for ORR of catalysts, linear sweep voltammetry (LSV) measurements in 0.1M KOH at room temperature was performed. As shown in Figure S6 (E), the half-wave potential of FeNx-CNTs electro-catalyst was 0.92V (versus RHE) in 0.1 mol L^-1^ KOH, showed a positive half-potential about 50 mV than that of commercial Pt/C.

**Preparation of QPPO ionomer**

QPPO was synthesized according to a typical procedure. Briefly, PPO (12 g) was dissolved in 100 mL of chlorobenzene to form a homogenous solution; then, NBS (4.27g) and AIBN (0.62 g) were added. The mixture was reacted at reflux conditions (135 °C) for 3 h. After cooling, the resulted solution was poured into 10-fold of ethanol to precipitate the product. The brominated PPO was filtered and washed several times and then dried under at 60 °C. Following, brominated PPO was dissolved in NMP and then added with excessive trimethylamine and stirred for 24 h at room temperature. The solution was poured into 10-fold excess of toluene, the precipitates (QPPO in Br^-^ form) were washed and collected. Finally, the Br^-^ was converted to OH^-^ by immersing in 1 mol L^-1^ NaOH for 24 h, and QPPO ionomer was obtained.


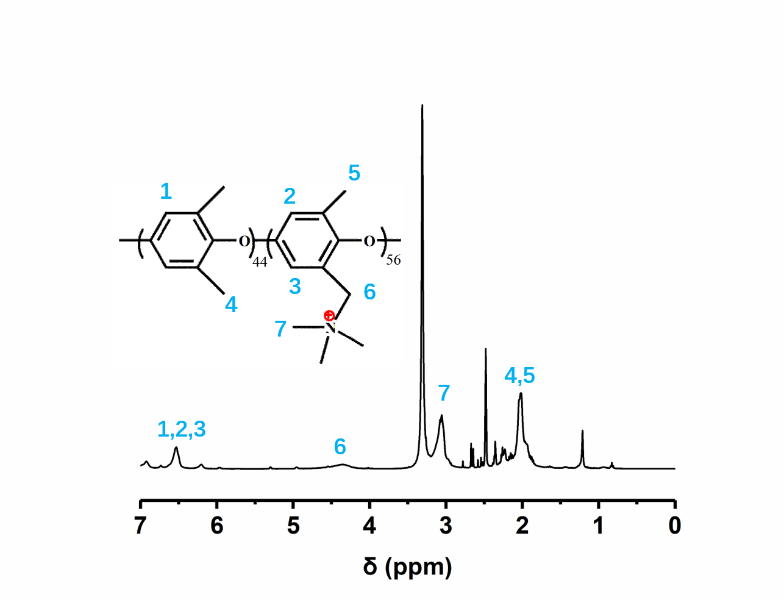


**Figure S7.** The chemical structure of QPPO ionomer used and its ^1^H NMR spectrum.

**Table S2**. Properties of QPPO ionomer

| Sample | IEC ^a^  (mmol g^-1^) | Water Uptake ^b^ (%) | Tensile Stress ^c^ (MPa) | σ @20 ℃^d^  (mS cm^-1^) | σ@80 ℃^d^  (mS cm^-1^) |
| --- | --- | --- | --- | --- | --- |
| QPPO | 1.64 | 22.3 | 30.2 | 33.0 | 88.2 |

^a^determined by titration; ^b^ measured at 20 ℃; ^c^ sample was fully hydrated; ^d^ OH^-^ conductivity


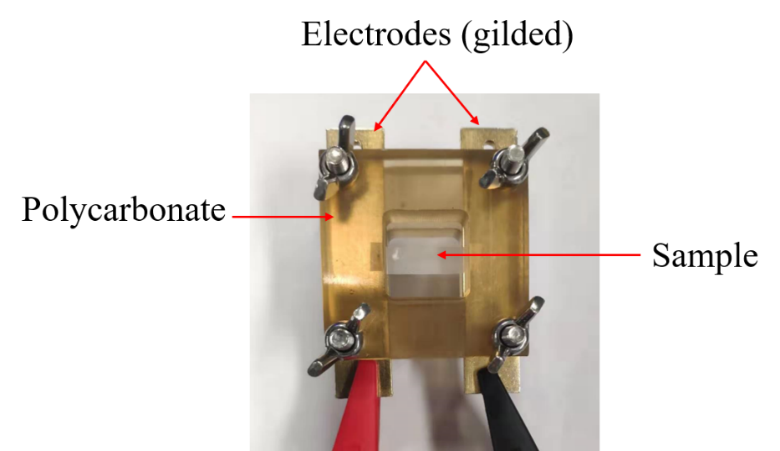


**Figure S8.** The conductivity testing fixture.

**References**

[1] Tondi, G., Tannin-based copolymer resins: synthesis and characterization by solid state ^13^C NMR and FT-IR spectroscopy. *Polymers*. 2017, 9 (12), 223, DOI: 10.3390/polym9060223.

[2] Wang, K.; Wu, Q.; Yan, X.; Liu, J.; Gao, L.; Hu, L.; Zhang, N.; Pan, Y.; Zheng, W.; He, G., Branched poly(ether ether ketone) based anion exchange membrane for H_2_/O_2_ fuel cell. *International Journal of Hydrogen Energy* 2019, 44 (42), 23750-23761, DOI: 10.1016/j.ijhydene.2019.07.080.

[3] Mansur, H. S.; Sadahira, C. M.; Souza, A. N.; Mansur, A. A. P., FTIR spectroscopy characterization of poly (vinyl alcohol) hydrogel with different hydrolysis degree and chemically crosslinked with glutaraldehyde. *Materials Science and Engineering: C* 2008, 28 (4), 539-548, DOI: 10.1016/j.msec.2007.10.088.

[4] Luo, Y.; Guo, J.; Wang, C.; Chu, D., Fuel cell durability enhancement by crosslinking alkaline anion exchange membrane electrolyte. *Electrochemistry Communications* 2012, 16 (1), 65-68, DOI: 10.1016/j.elecom.2012.01.005.

[5] Ge, X.; He, Y.; Guiver, M. D.; Wu, L.; Ran, J.; Yang, Z.; Xu, T., Alkaline anion-exchange membranes containing mobile ion shuttles. *Advanced Materials* 2016, 28 (18), 3467-72, DOI: 10.1002/adma.201506199, PMID: 26972938.

[6] Olsson, J. S.; Pham, T. H.; Jannasch, P., Poly(arylene piperidinium) hydroxide ion exchange membranes: synthesis, alkaline stability, and conductivity. *Advanced Functional Materials* 2018, 28 (2):1702758, DOI: 10.1002/adfm.201702758

[7] Pham, T. H.; Olsson, J. S.; Jannasch, P., N-Spirocyclic quaternary ammonium ionenes for anion-exchange membranes. *Journal of the American Chemical Society* 2017, 139 (8), 2888-2891, DOI: 10.1021/jacs.6b12944, PMID: 28192665.

[8] Tanaka, M.; Fukasawa, K.; Nishino, E.; Yamaguchi, S.; Yamada, K.; Tanaka, H.; Bae, B.; Miyatake, K.; Watanabe, M., Anion conductive block poly(arylene ether)s: synthesis, properties, and application in alkaline fuel cells. *Journal of the American Chemical Society* 2011, 133 (27), 10646-54, DOI: 10.1021/ja204166e, PMID: 21657275.

[9] Zeng, L.; Zhao, T. S.; An, L.; Zhao, G.; Yan, X. H., A high-performance sandwiched-porous polybenzimidazole membrane with enhanced alkaline retention for anion exchange membrane fuel cells. *Energy & Environmental Science* 2015, 8 (9), 2768-2774, DOI: 10.1039/C5EE02047F.

[10] Peng, X., Omasta T.J., Magliocca E., Wang L., Varcoe J.R., Mustain W.E.., Nitrogen-doped Carbon–CoO*_X_* Nanohybrids: a precious metal free cathode that exceeds 1.0 W cm^−2^ Peak power and 100 h life in anion-exchange membrane fuel cells. *Angewandte Chemie International Edition* 2019, 58 (4), 1046-1051, DOI: 10.1002/anie.201811099, PMID: 30414220.

[11] Zhu, Y.; Ding, L.; Liang, X.; Shehzad, M. A.; Wang, L.; Ge, X.; He, Y.; Wu, L.; Varcoe, J. R.; Xu, T., Beneficial use of rotatable-spacer side-chains in alkaline anion exchange membranes for fuel cells. *Energy & Environmental Science* 2018, 11, 12, 3472, 3479, DOI: 10.1039/C8EE02071J.

[12] Wang, Y.; Wang, G.; Li, G.; Huang, B.; Pan, J.; Liu, Q.; Han, J.; Xiao, L.; Lu, J.; Zhuang, L., Pt–Ru catalyzed hydrogen oxidation in alkaline media: oxophilic effect or electronic effect? *Energy & Environmental Science* 2015, 8 (1), 177-181, DOI: 10.1039/C4EE02564D.

[13] Zhu, L.; Zimudzi, T. J.; Li, N.; Pan, J.; Lin, B.; Hickner, M. A., Crosslinking of comb-shaped polymer anion exchange membranes via thiol–ene click chemistry. *Polymer Chemistry* 2016, 7 (14), 2464-2475, DOI: 10.1039/C5PY01911G.

[14] Wang, L., Magliocca E., Cunningham E.L., Mustain W.E., Poynton S.D., Escudero-Cid R., Nasef M.M., Ponce-González J., Bance-Souahli R., Slade R.C.T., Whelligan D.K., Varcoe J.R., An optimised synthesis of high performance radiation-grafted anion-exchange membranes. *Green Chemistry* 2017, 19 (3), 831-843, DOI: 10.1039/C6GC02526A.

[15] Zhu, L.; Yu, X.; Hickner, M. A., Exploring backbone-cation alkyl spacers for multi-cation side chain anion exchange membranes. *Journal of Power Sources* 2018, 375, 433-441, DOI: 10.1016/j.jpowsour.2017.06.020.

[16] Hou, J.; Liu, Y.; Ge, Q.; Yang, Z.; Wu, L.; Xu, T., Recyclable cross-linked anion exchange membrane for alkaline fuel cell application. *Journal of Power Sources* 2018, 375, 404-411, DOI: 10.1016/j.jpowsour.2017.06.073.

[17] Wang, Y.; Yang, Y.; Jia, S.; Wang, X.; Lyu, K.; Peng, Y.; Zheng, H.; Wei, X.; Ren, H.; Xiao, L.; Wang, J.; Muller, D. A.; Abruna, H. D.; Hwang, B. J.; Lu, J.; Zhuang, L., Synergistic Mn-Co catalyst outperforms Pt on high-rate oxygen reduction for alkaline polymer electrolyte fuel cells. *Nature Communications* 2019, 10 (1), 1506, DOI: 10.1038/s41467-019-09503-4, PMID: 30944328.

[18] Zhu, L.; Yu, X.; Peng, X.; Zimudzi, T. J.; Saikia, N.; Kwasny, M. T.; Song, S.; Kushner, D. I.; Fu, Z.; Tew, G. N.; Mustain, W. E.; Yandrasits, M. A.; Hickner, M. A., Poly(olefin)-based anion exchange membranes prepared using Ziegler–Natta polymerization. *Macromolecules* 2019, 52 (11), 4030-4041, DOI: 10.1021/acs.macromol.8b02756.

[19] Zhu, L.; Peng, X.; Shang, S. L.; Kwasny, M. T.; Zimudzi, T. J.; Yu, X.; Saikia, N.; Pan, J.; Liu, Z. K.; Tew, G. N.; Mustain, W. E.; Yandrasits, M.; Hickner, M. A., High performance anion exchange membrane fuel cells enabled by Fluoropoly(olefin) membranes. *Advanced Functional Materials* 2019, 29 (26), 1902059, DOI: 10.1002/adfm.201902059.

[20] Wang, L.; Peng, X.; Mustain, W. E.; Varcoe, J. R., Radiation-grafted anion-exchange membranes: the switch from low- to high-density polyethylene leads to remarkably enhanced fuel cell performance. *Energy & Environmental Science* 2019, 12 (5), 1575-1579, DOI: 10.1039/C9EE00331B.

[21] Assender, H. E.; Windle, A. H., Crystallinity in poly(vinyl alcohol) 2. Computer modelling of crystal structure over a range of tacticities. *Polymer* 1998, 39 (18), 4303-4312, DOI: 10.1016/S0032-3861(97)10297-X.
