# Supplementary figures and images for: Anion Exchange Membrane Based on Interpenetrating Polymer Network with Ultrahigh Ion Conductivity and Excellent Stability for Alkaline Fuel Cell
Source: Research (Wash D C). 2020 May 13;2020:4794706. doi: 10.34133/2020/4794706 (PMC7243038; doi:10.34133/2020/4794706)

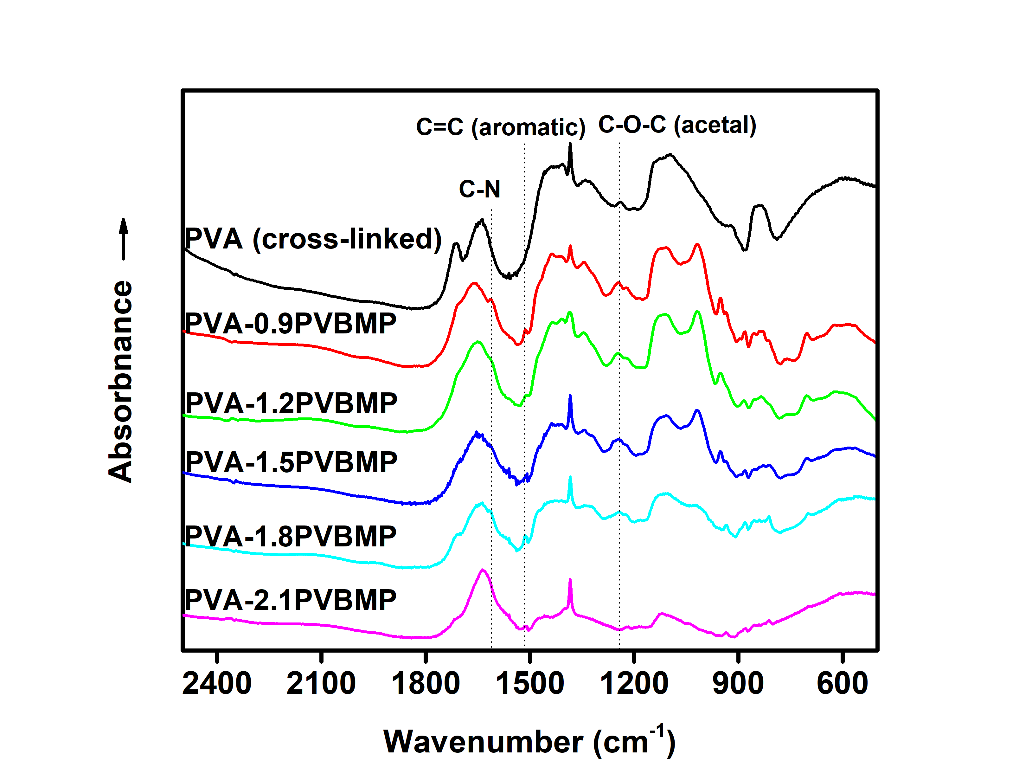

Supplement: Supplementary Materials — Figure S1: FTIR spectra of IPN AEMs and crosslinked PVA sample. Figure S2: SAXS data of crosslinked PVA and IPN AEMs samples; dry membranes were measured. Figure S3: the Arrhenius plots for calculation of apparent activation energy. Table S1: comparison of reported high-performance AEMs and this work. Figure S4: XRD patterns of crosslinked PVA and IPN AEMs. Figure S5: Proposed reason for the high alkaline resistance of IPN AEMs. Figure S6: SEM images of nanotubes-like FeNx-CNTs at (a) low magnification and (b) high magnification (c) and (d) TEM images of the FeNx-CNTs. (e) Polarization curves of FeNx-CNTs and commercial Pt/C in oxygen-saturated 0.1 M KOH at a rotating speed of 1600 rpm with a sweep rate of 10 mV s−1. Figure S7: the chemical structure of QPPO ionomer used and its 1H NMR spectrum. Table S2: properties of QPPO ionomer. Figure S8: the conductivity testing fixture. [file 4794706.f1.zip › 4794706.f1/S1.tif]

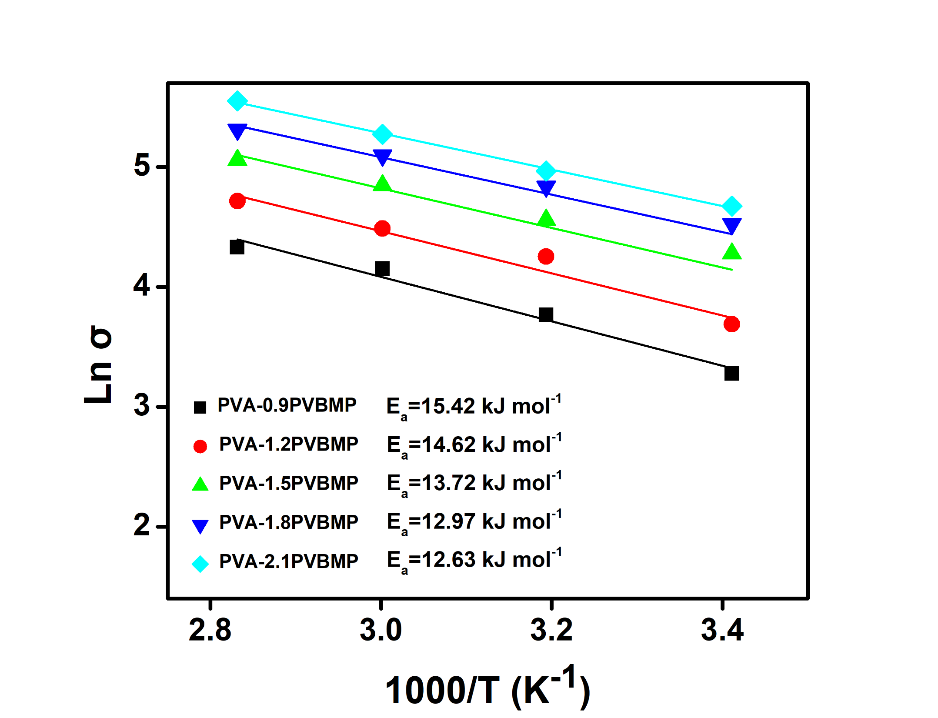

Supplement: Supplementary Materials — Figure S1: FTIR spectra of IPN AEMs and crosslinked PVA sample. Figure S2: SAXS data of crosslinked PVA and IPN AEMs samples; dry membranes were measured. Figure S3: the Arrhenius plots for calculation of apparent activation energy. Table S1: comparison of reported high-performance AEMs and this work. Figure S4: XRD patterns of crosslinked PVA and IPN AEMs. Figure S5: Proposed reason for the high alkaline resistance of IPN AEMs. Figure S6: SEM images of nanotubes-like FeNx-CNTs at (a) low magnification and (b) high magnification (c) and (d) TEM images of the FeNx-CNTs. (e) Polarization curves of FeNx-CNTs and commercial Pt/C in oxygen-saturated 0.1 M KOH at a rotating speed of 1600 rpm with a sweep rate of 10 mV s−1. Figure S7: the chemical structure of QPPO ionomer used and its 1H NMR spectrum. Table S2: properties of QPPO ionomer. Figure S8: the conductivity testing fixture. [file 4794706.f1.zip › 4794706.f1/S3.tif]

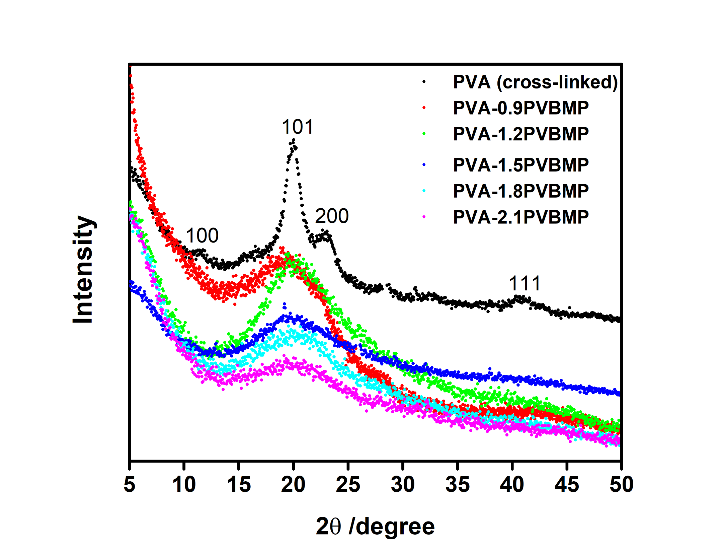

Supplement: Supplementary Materials — Figure S1: FTIR spectra of IPN AEMs and crosslinked PVA sample. Figure S2: SAXS data of crosslinked PVA and IPN AEMs samples; dry membranes were measured. Figure S3: the Arrhenius plots for calculation of apparent activation energy. Table S1: comparison of reported high-performance AEMs and this work. Figure S4: XRD patterns of crosslinked PVA and IPN AEMs. Figure S5: Proposed reason for the high alkaline resistance of IPN AEMs. Figure S6: SEM images of nanotubes-like FeNx-CNTs at (a) low magnification and (b) high magnification (c) and (d) TEM images of the FeNx-CNTs. (e) Polarization curves of FeNx-CNTs and commercial Pt/C in oxygen-saturated 0.1 M KOH at a rotating speed of 1600 rpm with a sweep rate of 10 mV s−1. Figure S7: the chemical structure of QPPO ionomer used and its 1H NMR spectrum. Table S2: properties of QPPO ionomer. Figure S8: the conductivity testing fixture. [file 4794706.f1.zip › 4794706.f1/S4.tif]

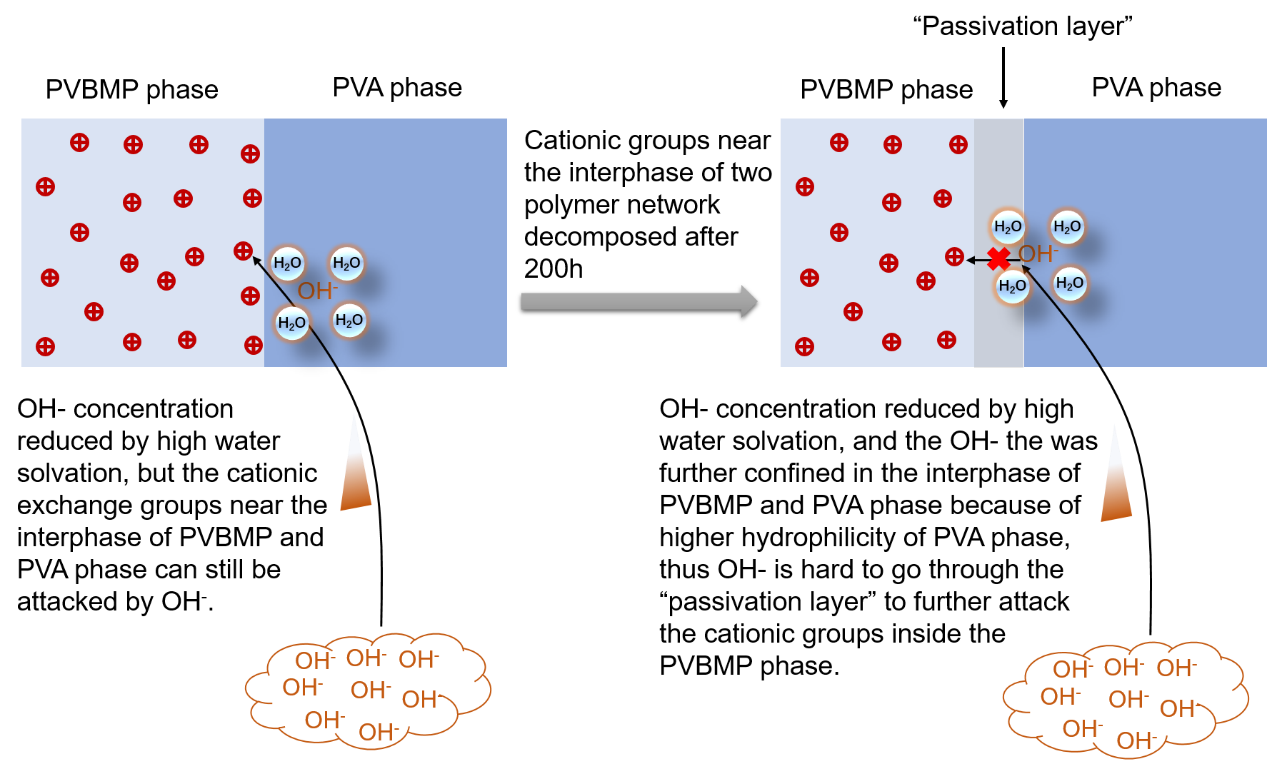

Supplement: Supplementary Materials — Figure S1: FTIR spectra of IPN AEMs and crosslinked PVA sample. Figure S2: SAXS data of crosslinked PVA and IPN AEMs samples; dry membranes were measured. Figure S3: the Arrhenius plots for calculation of apparent activation energy. Table S1: comparison of reported high-performance AEMs and this work. Figure S4: XRD patterns of crosslinked PVA and IPN AEMs. Figure S5: Proposed reason for the high alkaline resistance of IPN AEMs. Figure S6: SEM images of nanotubes-like FeNx-CNTs at (a) low magnification and (b) high magnification (c) and (d) TEM images of the FeNx-CNTs. (e) Polarization curves of FeNx-CNTs and commercial Pt/C in oxygen-saturated 0.1 M KOH at a rotating speed of 1600 rpm with a sweep rate of 10 mV s−1. Figure S7: the chemical structure of QPPO ionomer used and its 1H NMR spectrum. Table S2: properties of QPPO ionomer. Figure S8: the conductivity testing fixture. [file 4794706.f1.zip › 4794706.f1/S5.tif]

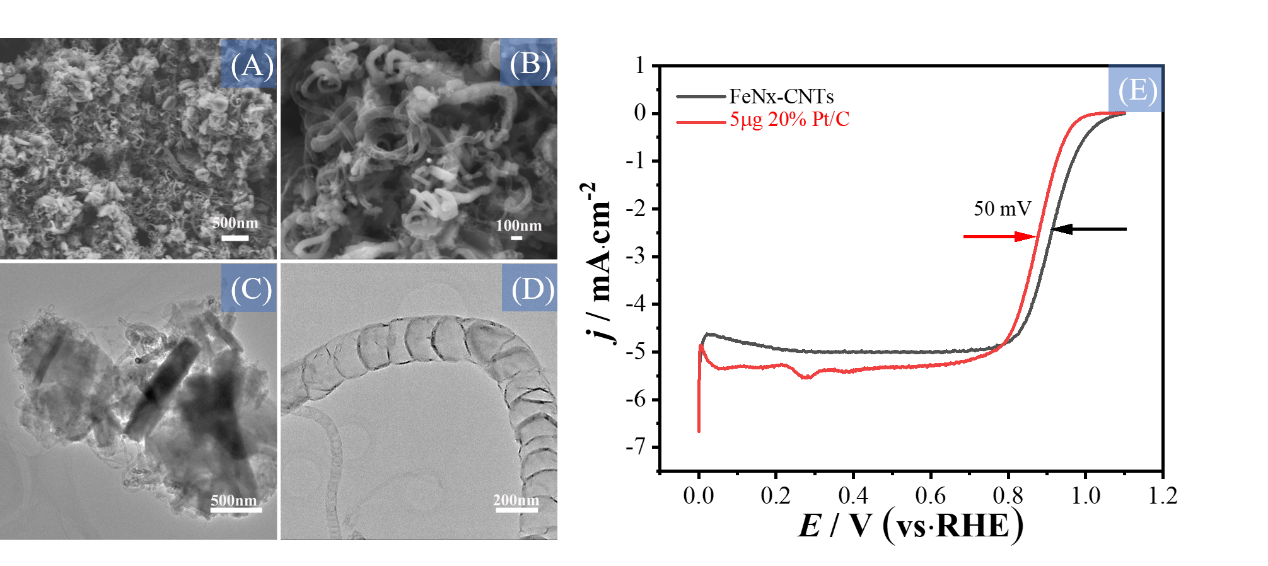

Supplement: Supplementary Materials — Figure S1: FTIR spectra of IPN AEMs and crosslinked PVA sample. Figure S2: SAXS data of crosslinked PVA and IPN AEMs samples; dry membranes were measured. Figure S3: the Arrhenius plots for calculation of apparent activation energy. Table S1: comparison of reported high-performance AEMs and this work. Figure S4: XRD patterns of crosslinked PVA and IPN AEMs. Figure S5: Proposed reason for the high alkaline resistance of IPN AEMs. Figure S6: SEM images of nanotubes-like FeNx-CNTs at (a) low magnification and (b) high magnification (c) and (d) TEM images of the FeNx-CNTs. (e) Polarization curves of FeNx-CNTs and commercial Pt/C in oxygen-saturated 0.1 M KOH at a rotating speed of 1600 rpm with a sweep rate of 10 mV s−1. Figure S7: the chemical structure of QPPO ionomer used and its 1H NMR spectrum. Table S2: properties of QPPO ionomer. Figure S8: the conductivity testing fixture. [file 4794706.f1.zip › 4794706.f1/S6.tif]

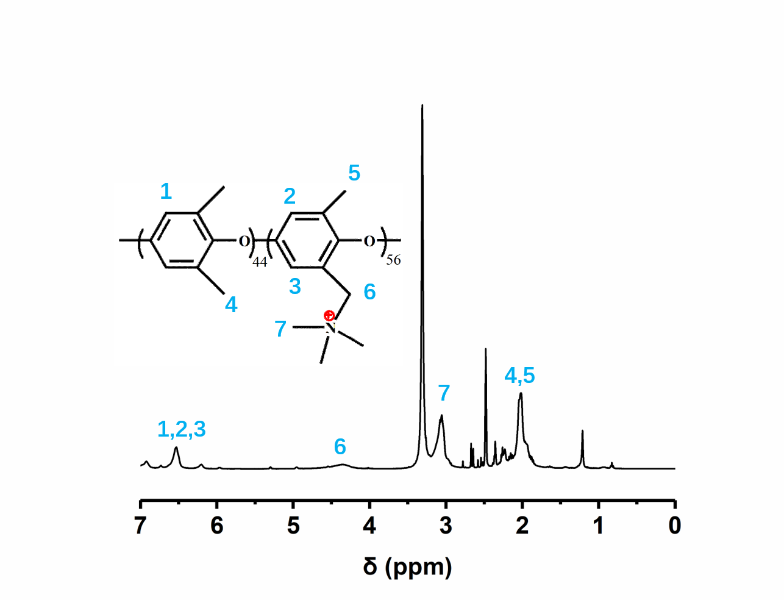

Supplement: Supplementary Materials — Figure S1: FTIR spectra of IPN AEMs and crosslinked PVA sample. Figure S2: SAXS data of crosslinked PVA and IPN AEMs samples; dry membranes were measured. Figure S3: the Arrhenius plots for calculation of apparent activation energy. Table S1: comparison of reported high-performance AEMs and this work. Figure S4: XRD patterns of crosslinked PVA and IPN AEMs. Figure S5: Proposed reason for the high alkaline resistance of IPN AEMs. Figure S6: SEM images of nanotubes-like FeNx-CNTs at (a) low magnification and (b) high magnification (c) and (d) TEM images of the FeNx-CNTs. (e) Polarization curves of FeNx-CNTs and commercial Pt/C in oxygen-saturated 0.1 M KOH at a rotating speed of 1600 rpm with a sweep rate of 10 mV s−1. Figure S7: the chemical structure of QPPO ionomer used and its 1H NMR spectrum. Table S2: properties of QPPO ionomer. Figure S8: the conductivity testing fixture. [file 4794706.f1.zip › 4794706.f1/S7.tif]

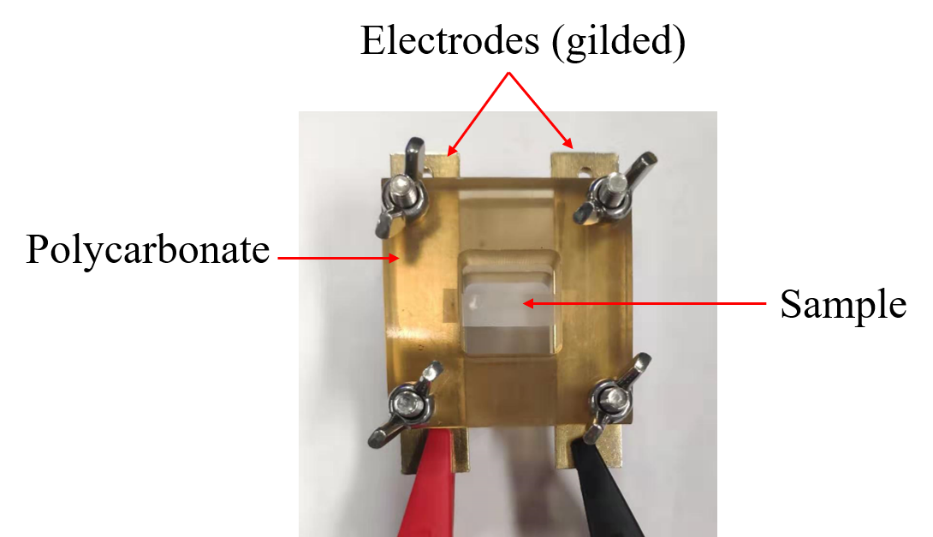

Supplement: Supplementary Materials — Figure S1: FTIR spectra of IPN AEMs and crosslinked PVA sample. Figure S2: SAXS data of crosslinked PVA and IPN AEMs samples; dry membranes were measured. Figure S3: the Arrhenius plots for calculation of apparent activation energy. Table S1: comparison of reported high-performance AEMs and this work. Figure S4: XRD patterns of crosslinked PVA and IPN AEMs. Figure S5: Proposed reason for the high alkaline resistance of IPN AEMs. Figure S6: SEM images of nanotubes-like FeNx-CNTs at (a) low magnification and (b) high magnification (c) and (d) TEM images of the FeNx-CNTs. (e) Polarization curves of FeNx-CNTs and commercial Pt/C in oxygen-saturated 0.1 M KOH at a rotating speed of 1600 rpm with a sweep rate of 10 mV s−1. Figure S7: the chemical structure of QPPO ionomer used and its 1H NMR spectrum. Table S2: properties of QPPO ionomer. Figure S8: the conductivity testing fixture. [file 4794706.f1.zip › 4794706.f1/S8.tif]
